# Correlation between Hashimoto’s thyroiditis and polycystic ovary syndrome: A systematic review and meta-analysis

**DOI:** 10.3389/fendo.2022.1025267

**Published:** 2022-10-31

**Authors:** Xiaojie Hu, Yuquan Chen, Yiting Shen, Siyuan Zhou, Wenting Fei, Yuxin Yang, Huafa Que

**Affiliations:** ^1^ Department of Surgery of Traditional Chinese Medicine, Longhua Hospital Shanghai University of Traditional Chinese Medicine, Shanghai, China; ^2^ Longhua Medical College, Shanghai University of Traditional Chinese Medicine, Shanghai, China; ^3^ Institute of Medical Information/Medical Library, Chinese Academy of Medical Sciences & Peking Union Medical College, Beijing, China

**Keywords:** polycystic ovary syndrome, Hashimoto’s thyroiditis, correlation, systematic review, meta-analysis

## Abstract

**Objective:**

A growing body of research suggests that patients with polycystic ovary syndrome (PCOS) may be at increased risk of developing Hashimoto’s thyroiditis (HT), and having both conditions can make the condition worse. However, current research views are not uniform. Therefore, to explore the link between PCOS and HT, we conducted this study.

**Methods:**

From the establishment of the database to August 2022, we searched 2 databases to study the correlation between Hashimoto’s and polycystic ovary syndrome. Two authors independently screened the articles for eligibility, and three authors extracted relevant data. Statistical analysis was performed using STATA16.0 software.

**Results:**

A total of 20 studies were included, including 7 case-control studies and 13 cross-sectional studies. A total of 13 countries and 7857 participants were embraced. Studies have demonstrated that both PCOS patients have an increased risk of HT, and meanwhile, HT patients also have an increased risk of PCOS compared with controls. The study also incorporated that the prevalence of HT in PCOS patients in India and Turkey was higher than in other countries, and the prevalence of HT in PCOS patients in South America was higher than in Asia and Europe.

**Conclusions:**

In conclusion, our study illustrates that there is a correlation between PCOS and HT, and it is necessary to further study the underlying mechanism between PCOS and HT. At the same time, it is of great significance to regularly screen PCOS patients for HT risk and HT patients for PCOS risk.

**Systematic Review Registration:**

https://www.crd.york.ac.uk/prospero/, identifier CRD 42022351168.

## Introduction

Polycystic ovary syndrome (PCOS) is one of the most common reproductive endocrine diseases in women of childbearing age, and is often clinically manifested by hyperandrogenemia, ovulatory dysfunction (ie, oligo- or anovulatory), and polycystic ovaries (1). Hashimoto’s thyroiditis (HT) is one of the most common thyroid disease which is frequently observed in young women (2, 3). Both PCOS and HT can induce the occurrence of related endocrine and metabolic diseases, increasing the risk of cardiovascular diseases and malignant tumors (4–6). Moreover, both diseases can occasion infertility in women of reproductive age (7, 8). Growing research evidence suggests that there may be an interaction between PCOS and HT. But their relationship remains controversial. Serin et al. displayed that HT can worsen the condition of PCOS patients (9). A meta-analysis of 1210 PCOS patients in 13 studies reported the association of polycystic ovary syndrome with autoimmune thyroid disease (10). However, new literature recently pointed out that the correlation between PCOS and HT is not strong.

Considering newly published studies in this field, as well as the limitations and selection bias of previous studies, updated systematic reviews and meta-analyses are needed to better clarify the link between PCOS and HT. Therefore, we elaborated a new meta-analysis aimed at assessing the association between PCOS and HT through a comprehensive search of the literature.

## Methods

### Registration

This review follows the Preferred Reporting Project for Systematic Reviews and Meta-Analysis (PRISMA) and is registered with PROSPERO (CRD 42022351168).

### Search strategy

A literature search was performed according to the literature search principles recommended in PRISMA. Two authors (Xiaojie Hu and Yuquan Chen) independently searched Pubmed and Embase databases, respectively, using a combination of subject headings and free words to search: “Hashimoto Disease” OR “Disease, Hashimoto” OR “Hashimoto Struma” OR “Hashimoto Thyroiditis” OR “Hashimoto Thyroiditides” OR “Thyroiditides, Hashimoto” OR “Thyroiditis, Hashimoto” OR “Hashimoto’s Syndrome” OR “Hashimoto Syndrome” OR “Hashimoto’s Syndromes” OR “Hashimotos Syndrome” OR “Syndrome, Hashimoto’s” OR “Syndromes, Hashimoto’s” OR “Hashimoto’s Struma” OR “Chronic Lymphocytic Thyroiditis” OR “Chronic Lymphocytic Thyroiditides” OR “Lymphocytic Thyroiditides, Chronic” OR “Lymphocytic Thyroiditis, Chronic” OR “Thyroiditides, Chronic Lymphocytic” OR “Thyroiditis, Chronic Lymphocytic” OR “Hashimoto’s Disease” OR “Disease, Hashimoto’s” OR “Hashimotos Disease” OR “Autoimmune thyroiditis” AND ““Polycystic Ovary Syndrome” OR “Ovary Syndrome, Polycystic” OR “Syndrome, Polycystic Ovary[“ OR “Stein-Leventhal Syndrome” OR “Stein Leventhal Syndrome” OR “Syndrome, Stein-Leventhal” OR “Sclerocystic Ovarian Degeneration” OR “Ovarian Degeneration, Sclerocystic” OR “Sclerocystic Ovary Syndrome” OR “Polycystic Ovarian Syndrome” OR “Ovarian Syndrome, Polycystic” OR “Polycystic Ovary Syndrome 1” OR “Sclerocystic Ovaries” OR “Ovary, Sclerocystic” OR “Sclerocystic Ovary”. The retrieval date was from the establishment of the database to August 2022, and all articles were published in English.

### Study selection criteria

The inclusion criteria for this study were as follows: (i) the study type must be observational; (ii) the subjects were patients with PCOS or HT; (iii) participants were regardless of race. Exclusion criteria were as follows: (i) case reports, reviews, and animal studies; (ii) studies with incomplete information, and the authors could not be contacted to obtain the required data. According to the inclusion and exclusion criteria, the two authors (Yiting Shen and Siyuan Zhou) screened literature in terms of the title and abstract, excluded the studies that did not meet the inclusion requirements, and finally screened out the articles that met the inclusion criteria. Differences in the review process were resolved by the addition of a third author (Huafa Que).

### Assessment of bias risk

The included studies were independently assessed by two authors using the Newcastle-Ottawa Scale (NOS) for methodological quality assessment (11). The scores of the included studies were all 7 points or above. Discuss and adjudicate disagreements were encountered by a third senior author (Huafa Que).

### Data extraction

For the included literature, we extracted the following information: first author, publication country, publication year, study design, diagnostic criteria, case sample, control sample, and total sample size. Data were extracted independently by three authors (Xiaojie Hu, Wenting Fei, and Yuxin Yang) and reviewed by one of the authors (Xiaojie Hu) for the accuracy of data extraction.

### Data analyses

For dichotomous data, we used OR or RR, 95% confidence interval (Cl). For continuous data, we used the weighted mean difference with 95% CI. OR is used to describe case-control studies and RR is used to describe cohort studies. Heterogeneity among included studies was assessed using the q-test and the squared value of I (12, 13). In the q-test, *p* < 0.10 or I² > 50% indicated the specific statistical significance of between-study heterogeneity, using a random-effects model. Conversely, the square of I is ≤50%, indicating that the heterogeneity among the included studies is small, and a fixed effect model can be used. Subgroup analyses and sensitivity analyses were used to explore the reasons for the heterogeneity. Egger’s test and funnel plot were used to analyze the possibility of publication bias. Therefore, STATA 16.0 software was used for statistical analysis.

### Ethical approval

The study did not involve vetting of participants and therefore did not require ethical approval.

## Result

### Study characteristics

**Figure 1:**
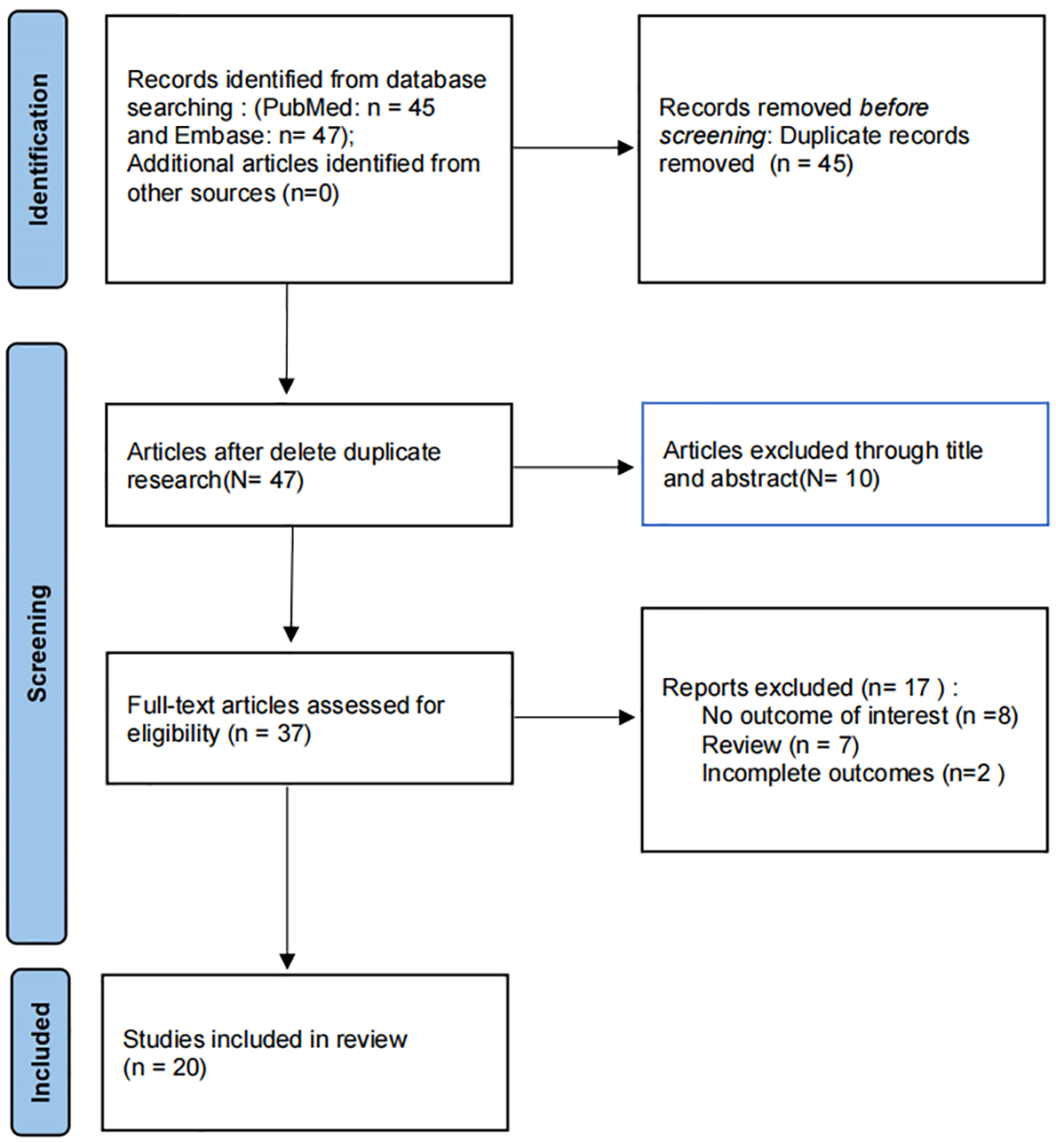
Flow diagram of systemic review procedure.

A preliminary search of 92 articles was carried out, and after the removal of duplicate entries, 37 articles were performed by full-text screening. Then, 17 articles were excluded. Therefore, a total of 20 studies were included in this study (**Figure 1**), including 7 case-control studies and 13cross-sectional studies. This study involved 13 countries, including 6 European studies, 12 Asian studies, and 2 South American studies, containing 3348 cases and 4509 control subjects. Rotterdam criteria were used as a diagnostic method for PCOS in 17 of the studies we included. Two studies utilized a non-Rotterdam diagnosis and one study did not report a diagnosis of PCOS. The diagnosis of HT in all included studies was based on thyroid autoimmunity (anti-TG, anti-TPO) (**Table 1**).

**Table 1:** Characteristics of included studies.

Study	Country	Year of publication	Study design	Diagnostic criteria	Total population	Sample size
PCOS preceding	HT diagnosis				cases; controls	cases; controls
Petrikova (14)	Slovak Republic	2015	Cross section	Rotterdam	64 cases; 68 controls	12 cases; 7 controls
Janssen (15)	Germany	2004	Cohort	non-	175 cases; 168 controls	47 cases; 14 controls
Kachuei (16)	Iran	2012	Case-control	Rotterdam	72 cases; 90 controls	22 cases; 25controls
Petríková (17)	Slovenska Republika	2012	Cross section	Rotterdam	64 cases; 68controls	12 cases; 7 controls
Sinha (18)	India	2013	Cross section	Rotterdam	80 cases; 80 controls	18 cases; 1 controls
Garelli (19)	Italy	2013	Cross section	Rotterdam	113 cases; 100 controls	30 cases; 8 controls
Novais (20)	Brazil	2015	Cross section	Rotterdam	65 cases; 65 controls	28 cases; 17 controls
Duran (21)	Turkey	2015	Cross section	Rotterdam	73 cases; 60 controls	23 cases; 14 controls
Arduc (22)	Turkey	2015	Cross section	Rotterdam	86 cases; 60 controls	19 cases; 3 controls
Calvar (23)	Argentina	2015	Case-control	Rotterdam	142 cases; 52 controls	27 cases; 7 controls
Yu (24)	China	2016	Case-control	Rotterdam	100 cases; 100 controls	25 cases; 2 controls
Yasar (25)	Turkey	2016	Case-control	Rotterdam	217 cases; 131 controls	47 cases; 23 controls
Arora (26)	India	2016	Cross section	Rotterdam	55 cases; 51 controls	21 cases; 8 controls
Adamska (27)	Poland	2020	Cross section	Rotterdam	A:67,B:30,C:28,D:16 141 cases; 88 controls	31 cases; 21 controls
Kim (28)	Korea	2022	Cross section	Rotterdam	104 cases; 238 controls	5 cases; 18 controls
Skrzynska (29)	Poland	2022	Cross section	non-	80 cases; 64 controls	18 cases; 9 controls
Al-Saab (30)	Syria	2014	Case-control	Rotterdam	56 cases; 30 controls	11 cases; 1 controls
Karaköse (31)	Turkey	2017	Cross section	Rotterdam	97 cases; 71 controls	39 cases; 11 controls
HT preceding PCOS diagnosis					cases; controls	cases; controls
Ganie (32)	India	2010	Case-control	Rotterdam	175 cases; 46 controls	82 cases; 20 controls
Ho (33)	China	2020	Case-control	NR	1332cases; 2664 controls	19 cases; 16 controls

### Study quality

The Newcastle Ottawa-Scale (NOS) tool was used to assess the quality of included studies. The NOS tool includes 8 items, each star representing one point. The scores of the included literature in this meta-analysis were all above 7 points, of which 12 kinds of literature scored 9 points, 7 literature scored 8 points, and 1 literature scored 7 points (**Table 2**). The included studies were at low risk of bias.

**Table 2:** Newcastle-Ottawa Scale for assessing the quality of nonrandomized studies.

First author	Year of publication	Selection	Score (Stars) Comparability	Outcome	Total Score
Petrikova (14)	2015	★★★★	★	★★★	8
Janssen (15)	2004	★★★★	★	★★★	8
Kachuei (16)	2012	★★★★	★★	★★★	9
Petríková (17)	2012	★★★★	★★	★★★	9
Sinha (18)	2013	★★★★	★★	★★★	9
Garelli (19)	2013	★★★★	★	★★★	9
Novais (20)	2015	★★★★		★★★	7
Duran (21)	2015	★★★★	★	★★★	8
Arduc (22)	2015	★★★★	★★	★★★	9
Calvar (23)	2015	★★★★	★★	★★★	9
Yu (24)	2016	★★★★	★★	★★★	9
Yasar (25)	2016	★★★★	★★	★★★	9
Arora (26)	2016	★★★★	★	★★★	8
Adamska (27)	2020	★★★★	★	★★★	8
Kim (28)	2022	★★★★	★	★★★	8
Skrzynska (29)	2022	★★★★	★★	★★★	9
Al-Saab (30)	2014	★★★★	★★	★★★	9
Karaköse (31)	2017	★★★★	★★	★★★	9
Ganie (32)	2010	★★★★	★★	★★★	9
Ho (33)	2020	★★★★	★	★★★	8

“★” means 1 point, the higher the number of “★” means the better the quality.

### Rate of HT in patients with PCOS

In this meta-analysis, 18 studies (3348 patients) described the prevalence of HT in PCOS patients, ranging from 4.81% to 40.21%, with a mean of 25.24% (**Table 1**). An Indian study and a Turkish study showed that the prevalence of HT in PCOS patients was significantly higher than in other studies (40.21% and 38.18%). If these studies were excluded as possible outliers, the mean prevalence of HT in PCOS patients was 22.12%. The prevalence of HT in the control group was only 13.52%. The prevalence of HT in PCOS patients was significantly higher than that in controls.

Ten Asian studies demonstrated that the average prevalence of HT in PCOS patients was 25.62%, 6 European studies indicated that the average prevalence of HT in PCOS patients was 22.57%, and 2 South American studies exhibited that the prevalence of HT in PCOS patients was 31.41%.

### Risk of HT in PCOS patients

**Figure 2:**
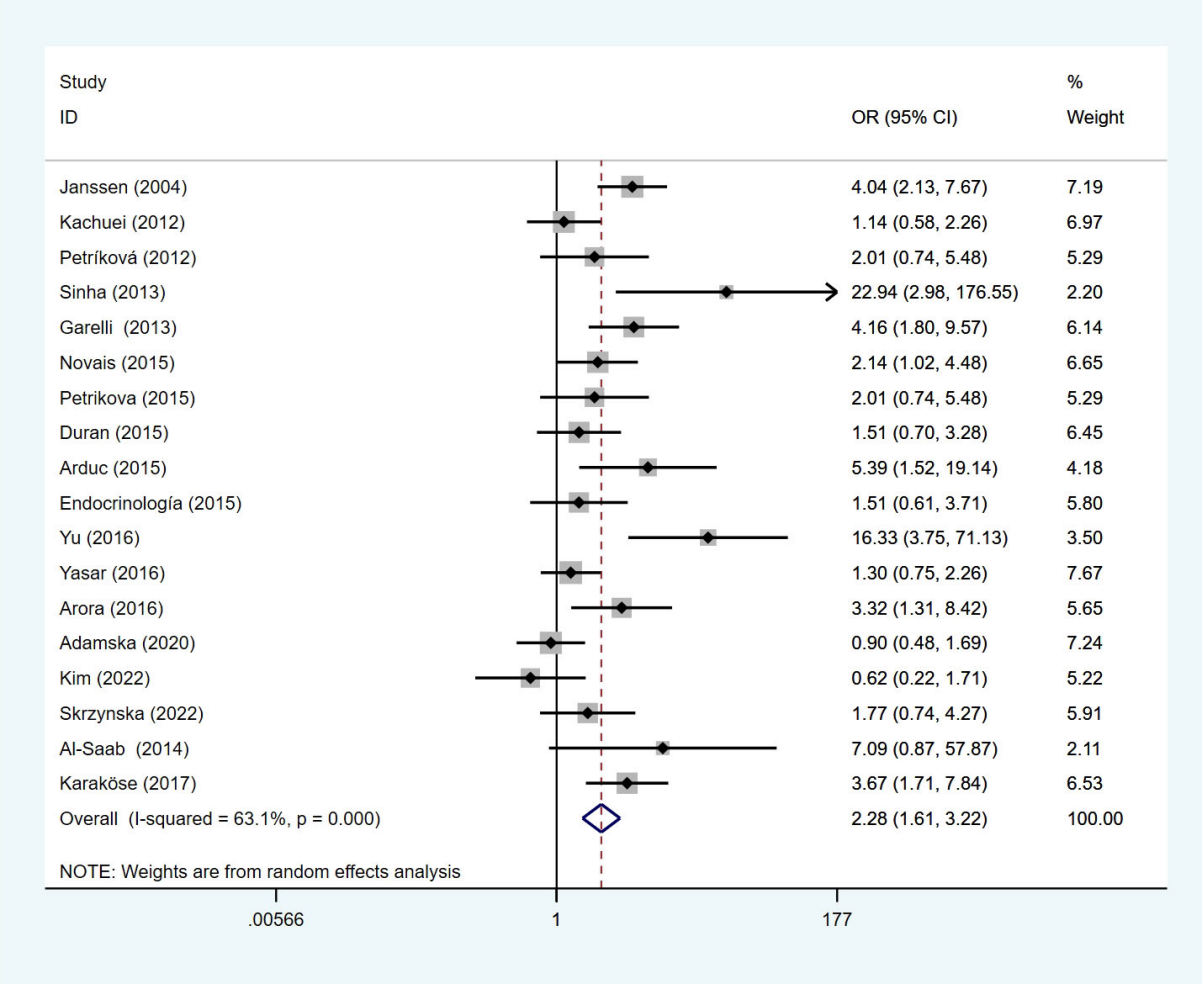
Forest plot of HT risk in PCOS patients and non-PCOS patients.

We performed a meta-analysis of 18 studies, and the results presented that PCOS patients had a higher risk of developing HT under a random-effects model (OR=2.28, 95%Cl=1.61-3.22, I^2 =^ 63.1%, p < 0.0001) (**Figure 2**). Heterogeneity analysis demonstrated that I²>50%, p<0.1, indicating that there was heterogeneity among the included studies, so sensitivity analysis and subgroup analysis were performed to find out the reasons for the heterogeneity. Sensitivity analysis indicated that the stability among the studies was good, the inclusion of literature was excluded in sequence, and the overall effect size on the results was not large.

**Figure 3:**
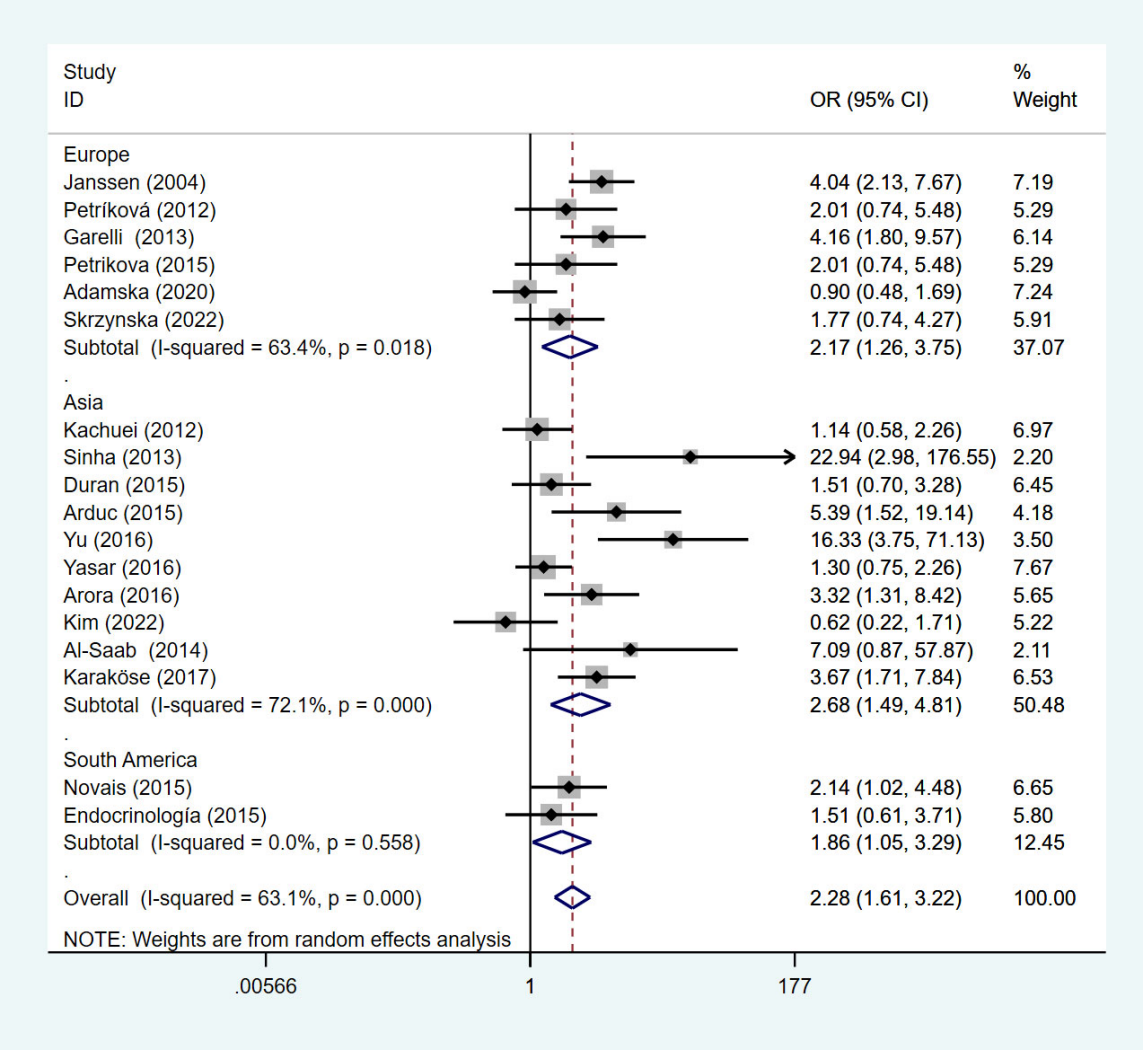
Forest plot for subgroup analysis of risk of HT in PCOS patients and non-PCOS patients by geographic location.

Under a random effects model, we performed a subgroup analysis by geographic location where the study was conducted. In the European, the OR of HT among PCOS patients was 2.17 (95%Cl=1.26-3.75, I^2^= 63.4%, p =0.018), 2.68 (95%Cl=1.49-4.81, I^2^= 72.1%, p <0.05) in Asian, and 1.86 (95%Cl=1.05-3.29, I^2^= 0%, p =0.558) in South American (**Figure 3**).

**Figure 4:**
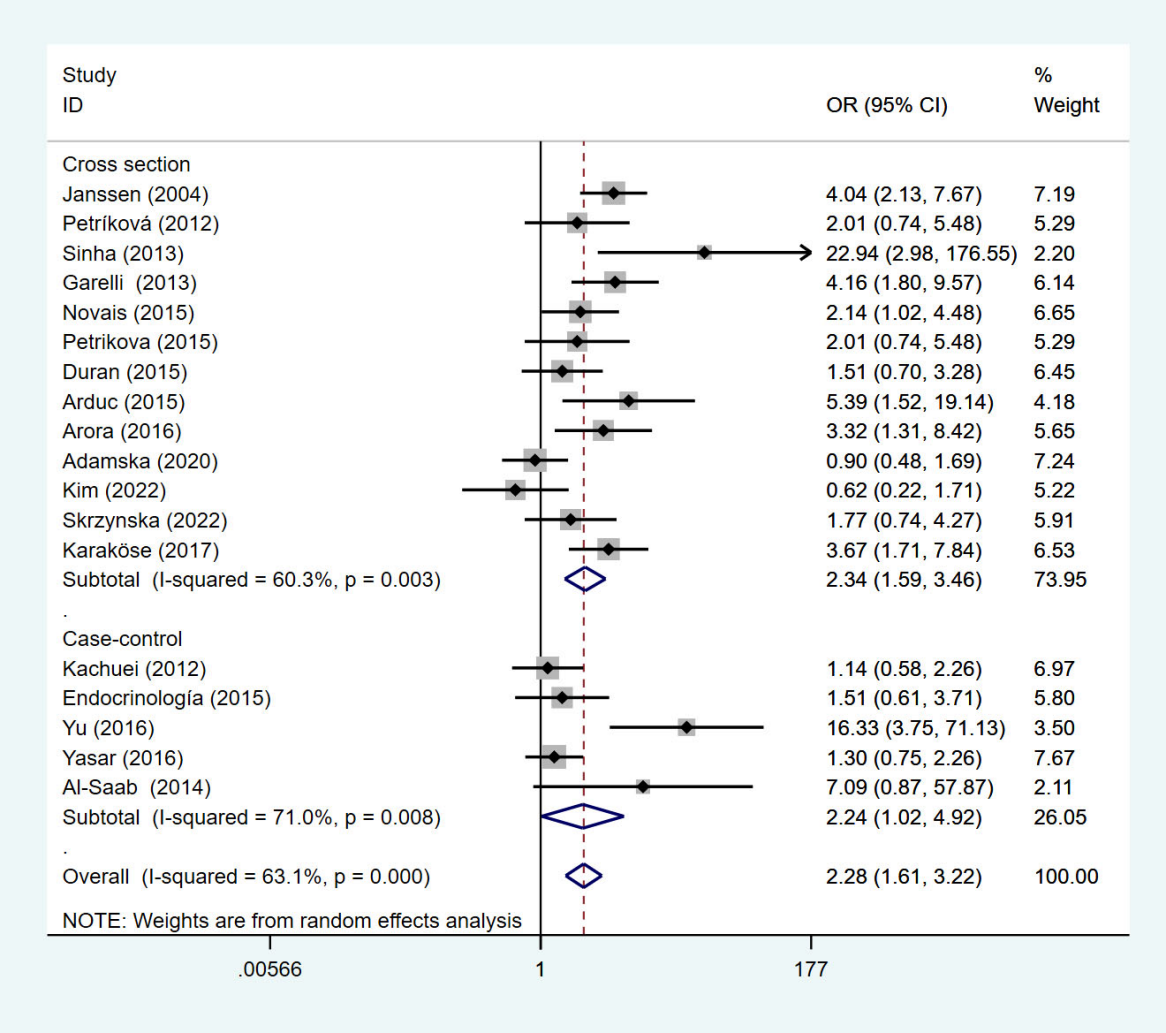
Forest plot for subgroup analysis of risk of HT in PCOS patients and non-PCOS patients by study type.

This meta-analysis of studies showed that compared with non-PCOS patients, Asian patients with PCOS were more likely to develop HT, followed by Europe, and lower in South America than in Europe. We divided the included studies into subgroup analyses according to the study type. Under the random effects model, the OR of the cross-sectional study was 2.34 (95%Cl=1.59-3.46, I2 = 60.3%, p =0.003), and the OR of the case-control study is 2.24 (95%Cl=1.02-4.92, I2 = 71.0%, p=0.008) (**Figure 4**).

### Risk of PCOS in patients with HT

**Figure 5:**
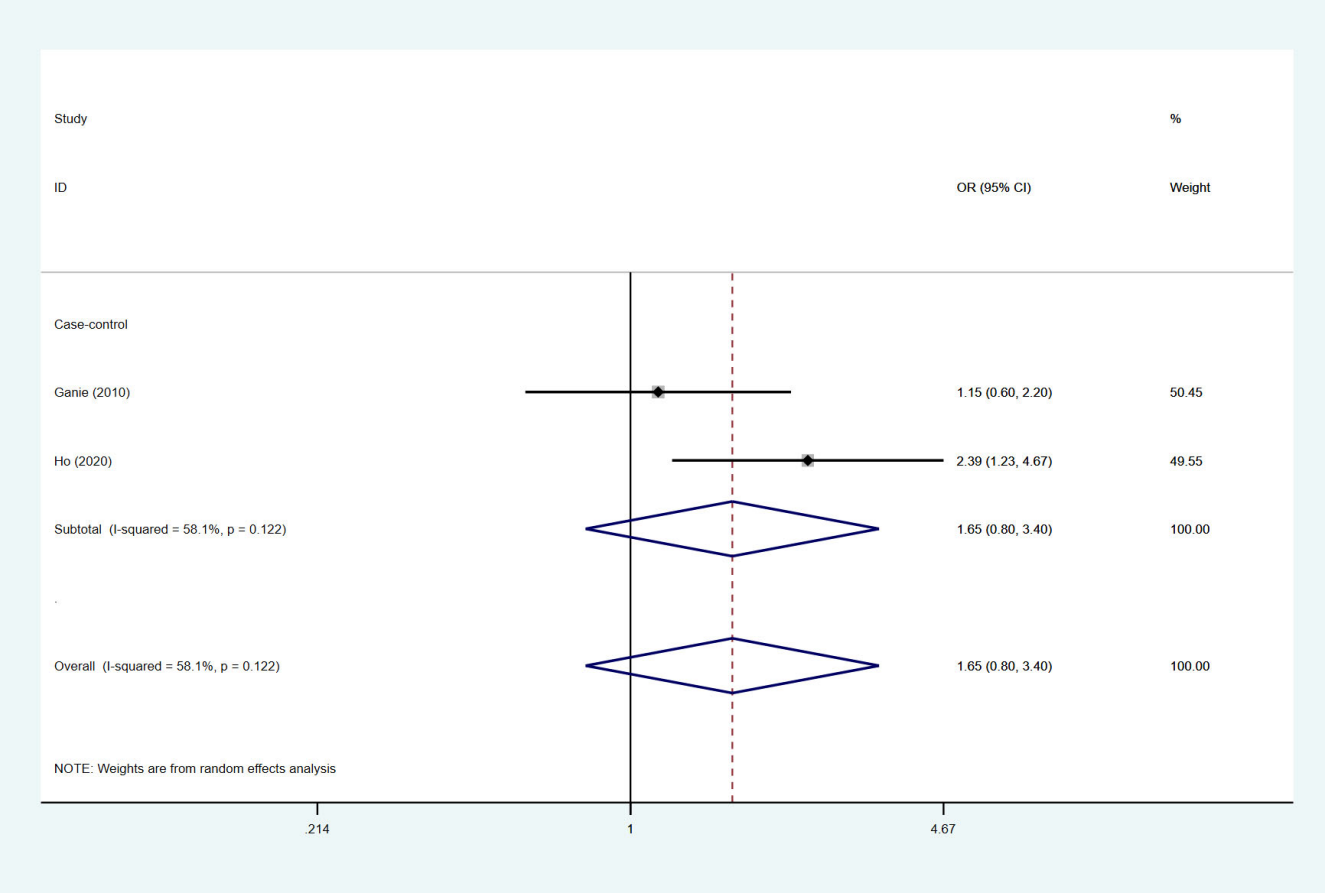
Forest map of PCOS risk in HT patients and non-HT patients.

A total of 2 studies in our included studies described the prevalence and risk of PCOS in patients with HT, and the mean prevalence of PCOS in patients with HT was 24.15%. Under the random effects model, the results of this meta-analysis showed that the OR was 1.65 (95%Cl=0.80-3.40, I2 = 58.1%, p=0.122) (**Figure 5**).

## Discussion

This study explored the correlation between PCOS and HT. This meta-analysis showed that patients with PCOS were more likely to develop HT than those without PCOS. At the same time, the study demonstrated that the risk of PCOS in HT patients is higher than that of non-HT patients. These findings suggest a significant association between PCOS and HT. This is consistent with previous research findings (10, 34).

At present, the pathogenesis of PCOS is not clear, but some studies have shown that the occurrence of PCOS may be related to genetic, metabolic, hormonal, and immune factors (35). The study pointed out that the PCOS-related gene for fibrillin 3(FBN3), gonadotropin-releasing hormone receptor (GnRHR), and CYP1B1 encoded to unction for estradiol hydroxylation may all be involved in the pathogenesis of PCOS and HT (36). PCOS-related fibrin 3 (FBN3) gene polymorphisms may be involved in the pathogenesis of HT and PCOS. Fibrin affects the activity of transforming growth factor beta (TGFβ). Multifunctional TGFβ is also a key regulator of immune tolerance by stimulating regulatory T cells (Treg), which can suppress excessive immune responses. The level of TGFβ1 was confirmed to be lower in HT and PCOS patients. TGFβ1 levels were lower in both HT and women with PCOS carrying the D19S884 allele 8 in the FBN3 gene, which may be consistent with unsuppressed autoimmune processes and the high incidence of HT in PCOS patients (37). Additionally, chronic low-grade inflammation plays an important role in promoting the occurrence and development of PCOS (38, 39). Excessive tumor necrosis factor caused by hyperglycemia exacerbates the metabolic and hormonal abnormalities of PCOS (40). In women with PCOS, advanced glycosylation end products (AGEs) and their receptors in the inflammatory and oxidative stress cascades have also been found to be overexpressed (41). Similarly, adipose tissue is involved in the pathogenesis of PCOS as a pro-inflammatory factor (42). The release of TNF-α and IL-6 from macrophages in adipose tissue is associated with the induction of insulin resistance (43). And hyperandrogenemia in PCOS can also lead to abnormal adipose function (44). Studies have evidenced an increased prevalence of various autoimmune diseases in PCOS patients (45). Seventy-eight percent of people with autoimmune disease are women and sex hormones appear to be involved in the immune response to infection in susceptible individuals (46). Autoimmune antibodies, including antinuclear antibodies (ANA), anti-smooth muscle antibodies, anti-histone antibodies, and anti-double-stranded DNA (dsDNA) antibodies, were significantly increased in PCOS patients (47, 48) Several studies have shown that TSH, TPO antibodies, and TG antibodies are also associated with immune-related factors, with thyroid-related factors increasing more than nonspecific autoimmune factors (14, 34). All evidence manifests a potential link between PCOS and HT. However, the specific various pathway mechanisms between these two diseases require further research in the future.

The study included multiple observational studies. We performed subgroup analyses by country and geographic location to reduce heterogeneity between studies. The results showed that there was a difference between the risk of HT in PCOS patients and the risk of HT in non-PCOS patients. There is a difference between the risk of PCOS in HT patients and the risk of PCOS in non-HT patients. Our findings indicate that the prevalence of HT in PCOS patients is highest in South America, followed by Europe, and the lowest in Asia. However, there were only 2 studies from South America and 10 studies from Europe. The number of published studies may affect the prevalence between continents, and the different methods of HT diagnosis in different regions will also cause a positive rate of HT diagnosis. Therefore, more and higher quality studies are needed to document the health management of PCOS patients and HT patients in the future.

Our research has the following advantages. We report an association between PCOS and HT, not only in PCOS patients at increased risk of HT but also in HT patients at increased risk of PCOS. At the same time, we also reported the risk and prevalence of HT in PCOS patients and the risk and prevalence of PCOS in HT patients across countries and continents. This was not mentioned in previous meta-analyses. Although there is heterogeneity in this meta-analysis, this may be related to differences in the regional environment, ethnicity, lifestyle, and HT diagnostic methods. Due to the lack of these details in the included studies, a more precise source of heterogeneity could not be found. Therefore, it is hoped that more and higher quality studies will explore the link between PCOS and HT in the future.

## Conclusions

In conclusion, our study demonstrates that there is an association between PCOS and HT and that PCOS patients in Indian and Turkish countries are at higher risk of developing HT, but more definitive conclusions need to be based on more high-quality studies. It is necessary to further study the underlying mechanism between PCOS and HT. Meantime, it is of great significance to regularly screen PCOS patients for HT risk and HT patients for PCOS risk.

## Data availability statement

The original contributions presented in the study are included in the article/supplementary material. Further inquiries can be directed to the corresponding author.

## Author contributions

XH and HQ co-designed this study. XH and YC drafted the research design. XH, YC, YS, and SZ search the database, delete duplicates, and filter according to the search subject. WF and YY extracted data and assessed the risk of bias. Data analysis was done by XH, YC, and HQ discussed with all members. Finally, the first draft is revised by XH. All authors contributed to the article and agreed to the submitted version.

## Funding

Construction project for National Regional Chinese medicine surgery diagnosis and treatment center (2018); Construction project for Shanghai Municipal Health Commission East China Area of TCM special disease alliance (2021); Construction project for Shanghai Municipal Health Commission key clinical speciality (shslczdzk03801); Construction project for Shanghai Municipal Health Commission inheritance and innovation team of the Shanghai-style Traditional Chinese Medicine (2021LPTD-001).

## Conflict of interest

The authors declare that the research was conducted in the absence of any commercial or financial relationships that could be construed as a potential conflict of interest.

## Publisher’s note

All claims expressed in this article are solely those of the authors and do not necessarily represent those of their affiliated organizations, or those of the publisher, the editors and the reviewers. Any product that may be evaluated in this article, or claim that may be made by its manufacturer, is not guaranteed or endorsed by the publisher.
